# RISK FACTORS FOR FALLS IN OLDER ADULTS WITH TYPE 2 DIABETES

**DOI:** 10.1093/geroni/igz038.1752

**Published:** 2019-11-08

**Authors:** Edgar R Vieira, Fabieli Fontes, Bartolomeu Filho, Fabricia Cavalcanti, Juliana Gazzola

**Affiliations:** 1 Department of Physical Therapy, Florida International University, Miami, Florida, United States; 2 Graduate Program in Physical Therapy, Federal University of Rio Grande do Norte, Natal, RN, Brazil; 3 Graduate Program in Physical Therapy, Federal University of Rio Grande do Norte, Natal, RN, Brazil, NATAL, Brazil; 4 Graduate Program in Physical Therapy, Federal University of Rio Grande do Norte, Natal, RN, Brazil, NATAL, Rio Grande do Norte, Brazil

## Abstract

The objective of this study was to evaluate the probability of the risk of falls in the older adults with type 2 diabetes. One-hundred and eleven older adults (age: 69±7 years) with type 2 diabetes participated in this cross-sectional observational study. The participants sociodemographics, physical function, cognitive status (Mini Mental State Exam – MEEM and Geriatric Depression Scale – GDS), balance (Mini BEST test), functional performance (WHODAS 2.0) and falls risk (Quick Screen Clinical Falls Risk Assessment – QuickScreen) were evaluated. The data was analyzed using the Kruskall-Wallis, Chi-square, and Fisher’s exact tests (p<0.05). Thirty percent of the participants had fallen during the previous 12 months, and 80% of the participants reported fear of falling. The average number of falls risks was 3.5±2. Increased number of falls risks were associated with lower educational level (p=0.005), poorer general health (p=0.001), vision impairment (p=0.017), higher number of diseases (p<0.0001), higher number of medications (p<0.0001), longer diabetes duration (p<0.0001), lower limb pain (p<0.0001), depression (p<0.001), worse functional performance (p<0.0001), and worse balance (p<0.0001). Older adults with type 2 diabetes with lower education, worse health and vision, greater number of diseases and medications, longer diagnosis of diabetes, lower limb pain, depressive symptoms, worse functional performance and balance presented more risks for falls.

